# Entropy Generation Optimization in Squeezing Magnetohydrodynamics Flow of Casson Nanofluid with Viscous Dissipation and Joule Heating Effect

**DOI:** 10.3390/e21080747

**Published:** 2019-07-30

**Authors:** Muhammad Zubair, Zahir Shah, Abdullah Dawar, Saeed Islam, Poom Kumam, Aurangzeb Khan

**Affiliations:** 1 Department of Mathematics, Abdul Wali Khan University, Mardan, Khyber, Pakhtunkhwa 23200, Pakistan; 2 Center of Excellence in Theoretical and Computational Science (TaCS-CoE), SCL 802 Fixed Point Laboratory, Science Laboratory Building, King Mongkut’s University of Technology Thonburi (KMUTT), 126 Pracha-Uthit Road, Bang Mod, Thrung Khru, Bangkok 10140, Thailand; 3 Department of Mathematics, Qurtuba University of Science and Information Technology, Peshawar 25000, Pakistan; 4 KMUTT-Fixed Point Research Laboratory, Room SCL 802 Fixed Point Laboratory, Science Laboratory Building, Department of Mathematics, Faculty of Science, King Mongkut’s University of Technology Thonburi (KMUTT), 126 Pracha-Uthit Road, Bang Mod, Thrung Khru, Bangkok 10140, Thailand; 5 KMUTT-Fixed Point Theory and Applications Research Group, Theoretical and Computational Science Center (TaCS), Science Laboratory Building, Faculty of Science, King Mongkut’s University of Technology Thonburi (KMUTT), 126 Pracha-Uthit Road, Bang Mod, Thrung Khru, Bangkok 10140, Thailand; 6 Department of Medical Research, China Medical University Hospital, China Medical University, Taichung 40402, Taiwan; 7 Department of Physics, Abdul Wali Khan University, Mardan 23200, Pakistan

**Keywords:** Casson fluid, magnetohydrodynamic (MHD), heat transfer, porosity, viscous dissipation, entropy, homotopy analysis method (HAM)

## Abstract

In this research article, the investigation of the three-dimensional Casson nanofluid flow in two rotating parallel plates has been presented. The nanofluid has been considered in steady state. The rotating plates have been considered porous. The heat equation is considered to study the magnetic field, joule heating, and viscous dissipation impacts. The nonlinear ordinary system of equations has been solved analytically and numerically. For skin friction and Nusslt number, numerical results are tabulated. It is found that velocity declines for higher values of magnetic and porosity parameter while it is heightened through squeezing parameter. Temperature is an enhancing function for Eckert number and nanoparticles volume fraction. Entropy generation is augmented with radiation parameter, Prandtl, and Eckert numbers. The Casson, porosity, magnetic field, and rotation parameters were reduced while the squeezing and suction parameters increased the velocity profile along *x*-direction. The porosity parameter increased the Bejan number while the Eckert and Prandtl numbers decreased the Bejan number. Skin friction was enhanced with increasing the Casson, porosity, and magnetic parameters while it decreased with enhancing rotation and squeezing parameters. All these impacts have been shown via graphs. The influences by fluid flow parameters over skin friction and Nusselt number are accessible through tables.

## 1. Introduction

In the modern world of science and technology, we need more development in the direction of the exhaustion of energy in engineering and industrial fields. Thus, for the exhaustion and transfer of heat, the study of nanofluids discloses extraordinary thermal conductivity and heat transfer coefficients compared to conventional fluids. These specific aspects of nanofluids make them appropriate for the succeeding generation of flow and heat transfer fluids. Therefore, the revolutionary nanofluids investigation has encouraged researchers and engineers all over the world. Problems related to the heat and mass transfer via nanofluid flow between two permeable walls/plates has remained under discussion since last few years. This is because heat and mass transfer have numerous applications in the industries of petroleum supplies, oil conveyance, and separation processes in chemical companies, etc. The advancement of power energy in technology is the essential objective of scientists and engineers. This is because of the extreme interest in cooling/heating in modern systems. Very high thermal conductivity of the nanofluid separates them from the other ordinary fluids as an ideal fluid. Since nanofluids have ideal thermal conductivity, they are used as cooling agents in computers, nuclear reactors, cancer therapy, to lower the level of cholesterol in blood, make surgeries safer, electronics, micro channels in defence sector, printing and press systems, food and drinks industries, as well as in oil, gas, and chemicals industries.

A key test for analysts is to upgrade the thermal conductivity applications of usual coolants corresponding to oil, ethylene glycol, and water, which have low thermal conductivity. Because of such inspiration, the first endeavor made by Choi [1] on this path was to encourage thermal conductivity of customary fluids including metallic nano-sized particles into a base fluid. Yu et al. [2] inspected the heat transmission and thermal conductivity through these nanofluid flows. Tyler et al. [3] investigated diamond-based nanofluid. Liu et al. [4] studied the carbon nanotubes nanofluid with thermal radiation. Ellahi et al. [5] fined the power series results of the nanofluids analytically. Nadeem et al. [6,7] examined the magnetohydrodynamic (MHD) nanofluids flow convection conditions. Shah et al. [8,9,10] inspected the nanofluids flows with Brownian, thermophoresis, and Hall impacts. Ramzan et al. [11] explored radiative flow of nanofluids by applying the impression of magnetic field. Sheikholeslami et al. [12] studied the flow of nanofluids via porous enclosure making an allowance for the magnetic effect. Besthapu et al. [13] explored the combined convection in nanofluid flow under the impressions of viscous dissipation and magnetic field influences. Dawar et al. [14] analyzed the nanofluid flow over a porous extending surface. In a rotating frame, Shah et al. [15] probed the Darcy–Forchheimer flow of nanofluid. Khan et al. [16] examined [15] considering the thermal radiation and magnetic field impacts. Khan et al. [17] evaluated two-dimensional flow of nanofluid over extending surface. Sheikholeslami [18] witnessed the convective nanofluid flow via porous medium by implementing effects of electric field. Sheikholeslami [19] studied the flow of nanofluid based on water with properties of Brownian motion. Dawar et al. [20] analyzed Darcy flow by using nanofluids past extending sheet. Ramzan et al. [21] observed the 3D flow of couple stress nanofluid. Sajid et al. [22] studied the fluid flow over a radially extending sheet. Attia et al. [23] examined the nanofluid flow through a porous median over radially extending sheet. 

The measure of disorder in a system or unavailable energy in a closed thermodynamically system is known as entropy. The entropy generation is derived from second law of thermodynamics. In 1980, the entropy production rate was introduced by Bejan [24]. Hayat et al. [25] investigated the nanomaterial fluid flow in a revolving system. Nouri et al. [26] studded the nanofluids flow through entropy generation considering the spherical heat source. Dalir et al. [27] scrutinized the Jeffrey nanofluid flow. Rashidi et al. [28] studied the steady MHD nanofluid flow in a rotating system. Das et al. [29] examined the MHD flow of nanofluids via porous medium. Seth et al. [30] calculated the Hall effect on Couette flow with entropy generation. Mkwizu et al. [31] numerically studied the Couette flow. Adesanya and Makinde [32] investigated the flow of couple stress nanofluid with entropy generation. Sheremet et al. [33] evaluated the nanofluid natural convection in a square cavity. In another article, Sheremet et al. [34] investigated the same fluid in wavy cavity. Alharbi et al. [35] evaluated the flow of nanofluid over porous extending sheet. Dawar et al. [36] examined the MHD carbon nanotubes nanofluid flow passing through rotating channels under the effects of viscous dissipation. Kumam et al. [37] studied the MHD nanofluid flow in rotating channels with entropy generation. Alain Portavoce et al. [38] explored transmission of atom in crystalline thin film. Oudina [39] explored heat transfer in Titania nanofluids in cylindrical annulus with discontinuous heat source. Oudina [40] studded MHD flow with natural convection between vertical coaxial cylinders. Reza et al. [41] explored nanofluids flow with MHD in a narrow channel with stretching walls. Jawad et al. [42] explored the MHD flow between two plates with different angle. Alkasassbeh et al. [43] investigated heat transfer by the fin attached to hybrid generator. Salim et al. [44] analyzed MHD jaffery suction flow through homotopy analysis method. Batti et al. [45] explored entropy production with MHD in nanofluid flow via porous stretching plate. Rashidi et al. [46] explored the entropy production in a single-slope solar still. Batti et al. [47] explored entropy production in entropy production with MHD during thermal radiation past a shrinking surface. Esfahani et al. [48] explored entropy production in nanofluid flow streaming past a wavy wall. For more study on entropy analysis, see [49,50,51,52,53,54,55,56,57].

In this research article, the investigation of the three-dimensional Casson nanofluid flow in two rotating parallel plates has been presented. The nanofluid has been considered in a steady state. The rotating plates have been considered porous. The heat equation is considered to study the magnetic field, joule heating, and viscous dissipation impacts. The nonlinear ordinary system of equations has been solved analytically. 

## 2. Problem Formulation

**Figure 1:**
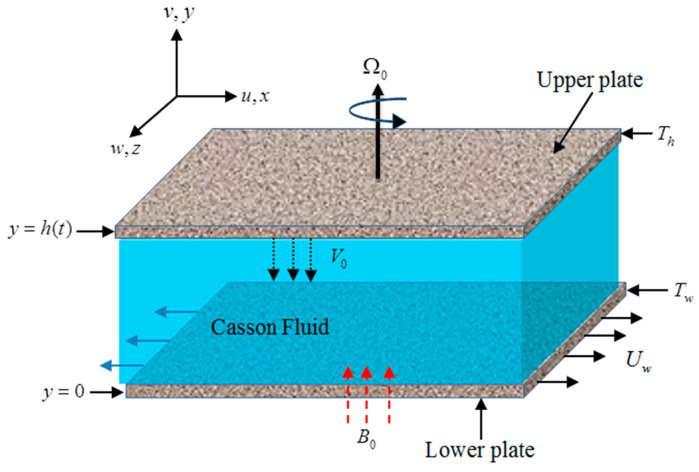
Physical sketch of the flow.

Here, we assumed 3D unsteady flow of Casson nanofluid to have viscous dissipation, joule heating, and entropy generation properties in two rotating parallel plates. Both plates are permeable. The suction velocity of the fluid via upper porous plate is $V_{0}$. The magnetic field’s strength $B=\frac{B_{0}}{\sqrt{1-st}}$ is also considered in the nanofluid flow. Darcy’s relation is implemented in order to study the nanofluid flow in a porous medium. The lower sheet is stretched with velocity $U_{w}=\frac{ax}{1-st}$ and is positioned at y=0. The upper sheet is squeezed towards the lower sheet with velocity $V_{h}=\frac{dh(t)}{dt}$ and is positioned at $h(t)=\sqrt{\frac{\upsilon_{nf}(1-st)}{a}}$ where a and s are the stretching and time parameters. The whole system rotates with angular velocity $\Omega_{0}$. The lower plate temperature is denoted by $T_{w}$ and the upper plate temperature is denoted by $T_{h}$. It is also supposed that $T_{w}>T_{h}$. Figure 1 shows the symmetric flow sketch of the nanofluids.

Nanofluid flow governing equations are [53,54,55,56]:

(1) $$\frac{\partial u}{\partial x}+\frac{\partial v}{\partial y}+\frac{\partial w}{\partial z}=0,$$

(2) $$\begin{array}{l} \frac{\partial u}{\partial t}+u\frac{\partial u}{\partial x}+v\frac{\partial u}{\partial y}+w\frac{\partial u}{\partial z}+2\frac{\Omega_{0}}{1-st}w+\frac{1}{\rho_{nf}}\frac{\partial P}{\partial x}\\ -\upsilon_{nf}\left(1+\frac{1}{\beta_{0}}\right)\left(\frac{\partial^{2}u}{\partial x^{2}}+\frac{\partial^{2}u}{\partial y^{2}}+\frac{\partial^{2}u}{\partial z^{2}}\right)+\left(\frac{\sigma_{nf}B_{0}^{2}}{\rho_{nf}}+\frac{\upsilon_{nf}}{\kappa^{*}}\right)\frac{u}{1-st}=0, \end{array}$$

(3) $$\frac{\partial v}{\partial t}+u\frac{\partial v}{\partial x}+v\frac{\partial v}{\partial y}+w\frac{\partial v}{\partial z}+\frac{1}{\rho_{nf}}\frac{\partial P}{\partial y}-\upsilon_{nf}\left(1+\frac{1}{\beta_{0}}\right)\left(\frac{\partial^{2}v}{\partial x^{2}}+\frac{\partial^{2}v}{\partial y^{2}}+\frac{\partial^{2}v}{\partial z^{2}}\right)+\frac{\upsilon_{nf}}{\kappa^{*}}\frac{v}{1-st}=0,$$

(4) $$\begin{array}{l} \frac{\partial w}{\partial t}+u\frac{\partial w}{\partial x}+v\frac{\partial w}{\partial y}+w\frac{\partial w}{\partial z}-2\frac{\Omega_{0}}{1-bt}u\\ -\upsilon_{nf}\left(1+\frac{1}{\beta_{0}}\right)\left(\frac{\partial^{2}w}{\partial x^{2}}+\frac{\partial^{2}w}{\partial y^{2}}+\frac{\partial^{2}w}{\partial z^{2}}\right)+\left(\frac{\sigma_{nf}B_{0}^{2}}{\rho_{nf}}+\frac{\upsilon_{nf}}{\kappa^{*}}\right)\frac{w}{1-st}=0, \end{array}$$

(5) $$\begin{array}{l} \frac{\partial T}{\partial t}+u\frac{\partial T}{\partial x}+v\frac{\partial T}{\partial y}+w\frac{\partial T}{\partial z}-\frac{\kappa_{nf}}{{\left(\rho C_{p}\right)}_{nf}}\left(\frac{\partial^{2}T}{\partial x^{2}}+\frac{\partial^{2}T}{\partial y^{2}}+\frac{\partial^{2}T}{\partial z^{2}}\right)\\ -\frac{\mu_{nf}}{{\left(\rho C_{p}\right)}_{nf}}\left[\begin{array}{l} 2{\left(\frac{\partial u}{\partial x}\right)}^{2}+2{\left(\frac{\partial v}{\partial y}\right)}^{2}+2{\left(\frac{\partial w}{\partial z}\right)}^{2}+{\left(\frac{\partial v}{\partial x}+\frac{\partial u}{\partial y}\right)}^{2}\\ +{\left(\frac{\partial u}{\partial z}+\frac{\partial w}{\partial x}\right)}^{2}+{\left(\frac{\partial v}{\partial z}+\frac{\partial w}{\partial y}\right)}^{2} \end{array}\right]\\ -\frac{\sigma_{nf}B_{o}^{2}}{{\left(\rho C_{p}\right)}_{nf}}\frac{\left(u^{2}+w^{2}\right)}{\left(1-st\right)}=0. \end{array}$$

Problem constrains at the boundary is of the form:

(6) $$\begin{array}{l} u=U_{w}=\frac{ax}{1-st},v=\frac{-V_{o}}{1-st},\;w=0,\;T=T_{w},\;at\;y=0,\\ u=0,\;v=-\frac{s}{2}\sqrt{\frac{\upsilon_{nf}}{a(1-st)}},w=0,\;T=T_{h},\;at\;y=h(t). \end{array}$$

The applied two forces are equal in measurement but reverse in direction, keeping the plates stretched. The lower plate is extended because of these equivalent forces in size and inverted direction, while the lower plate is squeezed down with velocity $V_{h}(t)$. $u=U_{w}=\frac{ax}{1-st}$ Illustrates the stretching velocity at y=0 while u=0 illustrates the free stream velocity at y=h(t). At the surface of the lower plate, there is a suction represented by $v=-\frac{V_{o}}{1-st}$ while at y=h(t) there is a squeezing velocity represented by $v=-\frac{s}{2}\sqrt{\frac{\upsilon_{nf}}{a(1-st)}}$. $T=T_{w}$ and $T=T_{h}$ characterize the constant temperature at the lower plate and rise of mercury at upper plate at y=0 and y=h(t), respectively. In the above equations u,v and w are the velocity components in their respective directions. $\kappa^{*}$ demonstrates the absorption coefficient or porosity of the medium and $\Omega_{0}$ define the angular velocity. Furthermore, $\mu_{nf}$ describes the nanofluid viscosity, $\rho_{nf}$ describes the nanofluid density, $\kappa_{nf}$ defines the nanofluid thermal conductivity, and ${\left(C_{p}\right)}_{nf}$ illustrates the nanofluid specific heat capacity, which are defined as

(7) $$\begin{array}{l} \frac{\mu_{nf}}{\mu_{f}}=\frac{1}{{\left(1-\Phi \right)}^{2.5}},\;\frac{\rho_{nf}}{\rho_{f}}=(1-\Phi )+\frac{\rho_{s}}{\rho_{f}}\Phi ,\;\frac{{\left(\rho C_{p}\right)}_{nf}}{{\left(\rho C_{p}\right)}_{f}}=(1-\Phi )+\frac{{\left(\rho C_{p}\right)}_{s}}{{\left(\rho C_{p}\right)}_{f}}\Phi ,\\ \frac{\kappa_{nf}}{\kappa_{f}}=\frac{\kappa_{s}+2\kappa_{f}-2\Phi (\kappa_{f}-\kappa_{s})}{\kappa_{s}+2\kappa_{f}+2\Phi (\kappa_{f}-\kappa_{s})},\;\frac{\sigma_{nf}}{\sigma_{f}}=1+\frac{3\left(\frac{\sigma_{s}}{\sigma_{f}}-1\right)\Phi }{\left(\frac{\sigma_{s}}{\sigma_{f}}+2\right)-\left(\frac{\sigma_{s}}{\sigma_{f}}-1\right)\Phi }. \end{array}$$

where $\Phi ,\mu_{f},\rho_{f},\kappa_{f},\sigma_{f},{\left(C_{p}\right)}_{f},s,f$, and nf indicate the nanoparticle volume fraction, dynamic viscosity, density, thermal conductivity, electric conductivity, specific heat capacity, solid nanoparticles, base fluid, and nano-fluid, respectively. 

The transformation variables are demarcated:

(8) $$\begin{array}{l} u=U_{w}f^{\prime }(\xi ),\;v=-\sqrt{\frac{a\upsilon_{nf}}{1-st}}f(\xi ),\;w=U_{w}g(\xi ),\\ \theta (\xi )=\frac{T-T_{h}}{T_{w}-T_{h}},\;h(t)=\sqrt{\frac{\upsilon_{nf}(1-st)}{a}},\;\xi =\frac{y}{h(t)}. \end{array}$$

Obviously, Equation (1) satisfies, and Equations (2)–(6) becomes:

(9) $$\left(1+\frac{1}{\beta_{0}}\right)f^{iv}-f^{\prime }f^{\prime \prime }+ff^{\prime \prime \prime }-\frac{\beta }{2}\left(3f^{\prime \prime }+\xi f^{\prime \prime \prime }\right)-2\alpha g^{\prime }-\frac{1}{N_{1}}\left(N_{2}M+N_{3}\lambda \right)f^{\prime \prime }=0,$$

(10) $$\left(1+\frac{1}{\beta_{0}}\right)g^{\prime \prime }+fg^{\prime }-f^{\prime }g-\beta \left(g+\frac{\xi }{2}g^{\prime }\right)+2\alpha f^{\prime }-\frac{1}{N_{1}}\left(N_{2}M+N_{3}\lambda \right)g=0,$$

(11) $$\frac{N_{1}N_{4}}{N_{3}N_{5}}\frac{1}{\mathrm{Pr}}\theta^{\prime \prime }+\left(f-\frac{\beta }{2}\xi \right)\theta^{\prime }+\frac{1}{N_{5}}\left[N_{3}E_{c}\left(4{f^{\prime }}^{2}+g^{2}\right)+E_{d}\left\{\begin{array}{l} N_{1}\left({g^{\prime }}^{2}+2{f^{\prime \prime }}^{2}\right)\\ +N_{2}M\left({f^{\prime }}^{2}+g^{2}\right) \end{array}\right\}\right]=0.$$

Satisfying the succeeding boundary conditions:

(12) $$\begin{array}{l} f^{\prime }=1,\;f=\delta ,\;g=0,\;\theta =1,\;\mathrm{at}\;\;\xi =0,\\ f^{\prime }=0,\;f=\frac{\beta }{2},\;g=0,\;\theta =0\;\mathrm{at}\;\xi =1. \end{array}$$

Here the following ratios are defined between the nanofluid and base fluid. $N_{1}=\frac{\rho_{nf}}{\rho_{f}}$, $N_{2}=\frac{\sigma_{nf}}{\sigma_{f}}$, $N_{3}=\frac{\mu_{nf}}{\mu_{f}}$, $N_{4}=\frac{\kappa_{nf}}{\kappa_{f}}$, and $N_{5}=\frac{{\left(\rho C_{p}\right)}_{nf}}{{\left(\rho C_{p}\right)}_{f}}$ define the density, electric conductivity, dynamic viscosity, thermal conductivity, and specific heat capacitance.

Also, $\beta_{o}$ clarifies the Casson parameter, $M=\frac{\sigma_{f}B_{o}^{2}}{a\rho_{f}}$ indicates the magnetic field, $\alpha =\frac{\Omega_{o}}{a}$ demonstrates the rotation parameter, $\beta =\frac{s}{a}$ displays the squeezing parameter, $\delta =\frac{V_{o}}{ah}$ defines the suction parameter, $\lambda =\frac{\upsilon_{f}}{a\kappa^{*}}$ determines the porosity parameter, $\mathrm{Pr}=\frac{\mu_{f}{\left(c_{p}\right)}_{f}}{\kappa_{f}}$ shows the Prandtl number, and $Ec=\frac{\upsilon_{f}^{2}}{{\left(c_{p}\right)}_{f}(T_{w}-T_{h})h^{2}}$ and $Ed=\frac{U_{w}^{2}}{{\left(c_{p}\right)}_{f}(T_{w}-T_{h})}$ are local Eckert numbers. 

The skin friction and Nusselt number of the nanofluid can be characterized as:

(13) $$C_{fx}=\frac{-2\tau_{w}}{\rho_{nf}U_{w}^{2}},\;Nu_{x}=\frac{xq_{w}}{\kappa_{nf}\left(T_{w}-T_{h}\right)},$$

where $\tau_{w}$ defines wall share stress and $q_{w}$ defines heat flux. Mathematically, we have:

(14) $$\tau_{w}={\mu_{nf}\left(\frac{\partial v}{\partial x}+\frac{\partial u}{\partial y}\right)|}_{y=0},\;{q_{w}=-\kappa_{nf}\frac{\partial T}{\partial y}|}_{y=0},$$

Substituting Equation (14) in Equation (13), in a simple way, is

(15) Cfx(Rex)=−2N3N1f″(0),Nux(Rex)=−N4N3N1θ′(0),

where Rex(=xUwυf) defines the Reynolds number.

## 3. Entropy Analysis

The rate of entropy production ($E_{G}$) is obtained as [47,48]:

(16) $$\begin{array}{l} E_{G}=\frac{k_{nf}}{T_{h}^{2}}\left[{\left(\frac{\partial T}{\partial x}\right)}^{2}+{\left(\frac{\partial T}{\partial y}\right)}^{2}+{\left(\frac{\partial T}{\partial z}\right)}^{2}\right]+\frac{\sigma_{nf}B_{o}^{2}}{{\left(\rho C_{p}\right)}_{nf}}\left(u^{2}+w^{2}\right)\\ +\frac{\mu_{nf}}{T_{h}}\left[\begin{array}{l} 2{\left(\frac{\partial u}{\partial x}\right)}^{2}+2{\left(\frac{\partial v}{\partial y}\right)}^{2}+2{\left(\frac{\partial w}{\partial z}\right)}^{2}\\ +{\left(\frac{\partial v}{\partial x}+\frac{\partial u}{\partial y}\right)}^{2}+{\left(\frac{\partial u}{\partial z}+\frac{\partial w}{\partial x}\right)}^{2}+{\left(\frac{\partial v}{\partial z}+\frac{\partial w}{\partial y}\right)}^{2} \end{array}\right]+\frac{\mu_{nf}}{T_{h}\kappa^{*}(1-st)}\left(u^{2}+v^{2}+w^{2}\right). \end{array}$$

Using Equations (8) and (16) reduced as:

(17) EG=EGO[N4θ′2+TcPrN32N1N4{Ec(4f′2+g2)+N1EdN3(f″2+g′2)+N2MEdN3(f′2+g2)+λEd(f′2+N3N1Rexf2+g2)}],

where $E_{G}{}_{{}_{o}}\left(=\frac{\kappa_{f}(T_{w}-T_{h})}{T_{h}^{2}h^{2}}\right)$ describes the characteristics entropy analysis quotient while $T_{c}=\frac{T_{h}}{T_{w}-T_{h}}$ defines the temperature ratio. 

The entropy generation rate is reduced as:

(18) Ns=EGEGo=[N4θ′2+TcPrN32N1N4{Ec(4f′2+g2)+N1EdN3(f″2+g′2)+N2MEdN3(f′2+g2)+λEd(f′2+N3N1Rexf2+g2)}].

In components form, Equation (18) is expressed as:

(19) Ns=Nh+Nf+Nj+Np.

The complete volumetric entropy production is demarcated as:

(20) $$E_{G_{total}}=\int_{0}^{h}E_{G}dy.$$

The non-dimensional total entropy generation is reduced as:

(21) Nstotal=∫0hNsdy=∫01[N4θ′2+TcPrN32N1N4{Ec(4f′2+g2)+N1EdN3(f″2+g′2)+N2MEdN3(f′2+g2)+λEd(f′2+N3N1Rexf2+g2)}]dξ.

### 3.1. Bejan Number

The Bejan number shows the quotient of entropy production rate for heat transfer to entire entropy production. It is characterized as:

(22) $$Be=\frac{Nh}{Ns}.$$

The Bejan number permanently lies in the range of 0 and 1. The Nh falls down in the region $0\leq Be<\frac{1}{2}$. The Nf+Nj+Np dominates in the region 0.5<Be≤1.0. For Be=0.5 in cooperation, the properties are balanced.

### 3.2. Solution by HAM

To solve the proposed model by analytical method called HAM [49,50,51,52], the initial supposition for Equations (9)–(11) are supposed as:

(23) $$f_{0}(\xi )=\xi -2\xi^{2}+\xi^{3}+3\delta \xi^{2}-2\delta \xi^{3},\;g_{0}(\xi )=0,\;\theta_{0}(\xi )=1-\xi .$$

The linear operators are defined as:

(24) $$L_{f}(f)=f^{iv},\;L_{g}(g)=g^{\prime \prime },\;L_{\theta }(\theta )=\theta^{\prime \prime }.$$

The initial solutions are:

(25) $$L_{f}(k_{1}+k_{2}\xi +k_{3}\xi^{2}+k_{4}\xi^{3})=0,\;L_{g}(k_{5}+k_{6}\xi )=0,\;L_{\theta }(k_{7}+k_{8}\xi )=0.$$

Here, $\sum_{n=1}^{8}k_{n},$ where n=1, 2, 3, … are random constants.

### 3.3. HAM Convergence

**Figure 2:**
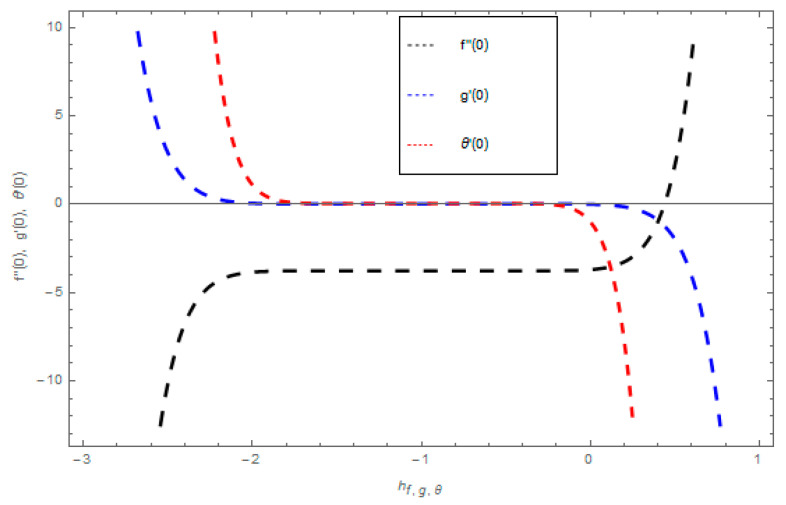
The combined ℏ curves for velocities $f^{\prime }\left(\xi \right)\;\mathrm{and}\;g\left(\xi \right)$ and temperature θ(ξ) profiles.

When we compute the series solutions of the velocity and temperature functions in order to use HAM, the assisting parameters $\hbar_{f,g,\theta }$ appear. These assisting parameters are responsible for adjusting the convergence of these solutions. The combined ℏ− curve of $f^{\prime \prime }\left(0\right),g^{\prime }\left(0\right)$ and θ(0) at 10th-order approximations are plotted in Figure 2 for different values of the embedding parameter. The combined ℏ− curve consecutively displays the valid region.

## 4. Results and Discussion

This segment treaties with the physical impacts of concerned factors of the nanofluid flow on velocity profiles $\left(f^{\prime }\left(\xi \right),f\left(\xi \right)\right)$, temperature profile θ(ξ), entropy production Ns(ξ), Bejan number Be(ξ), skin friction $C_{fx}$, and Nusselt number $Nu_{x}$. These parameters include Casson-$\beta_{o}$, Magnetic field-M, squeezing-β, porosity-λ, suction-δ, rotation-α, Prandtl number-Pr, and Eckert numbers-Ec,Ed.

**Figure 3:**
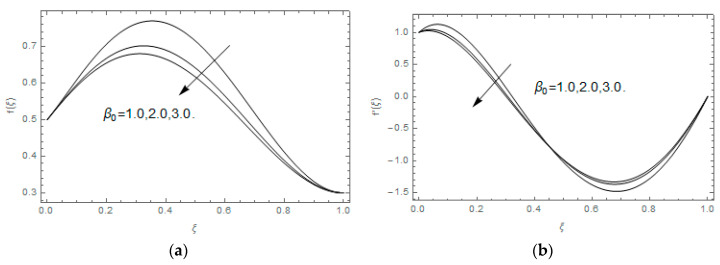
(**a**,**b**): Impression of $\beta_{o}$ on f(ξ) and $f^{\prime }\left(\xi \right)$.

**Figure 4:**
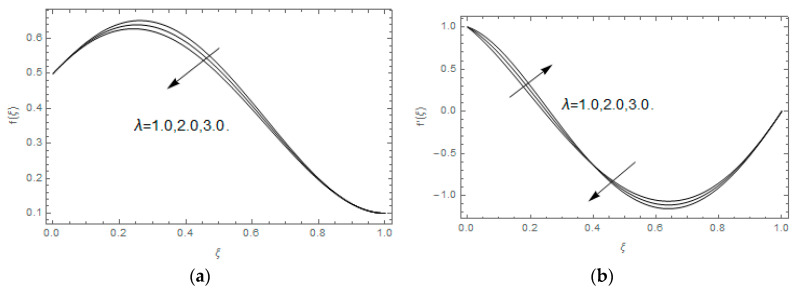
(**a**,**b**): Impression of λ on f(ξ) and $f^{\prime }\left(\xi \right)$.

**Figure 5:**
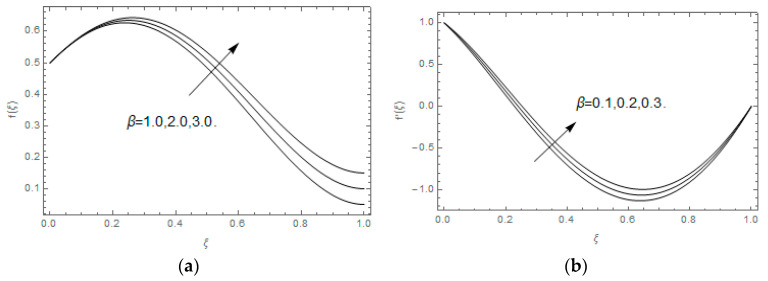
(**a**,**b**): Impression of β over f(ξ) and $f^{\prime }\left(\xi \right)$.

**Figure 6:**
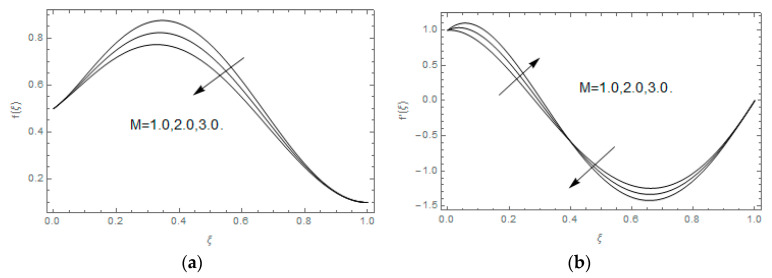
(**a**,**b**): Impact of M on f(ξ) and $f^{\prime }\left(\xi \right)$.

**Figure 7:**
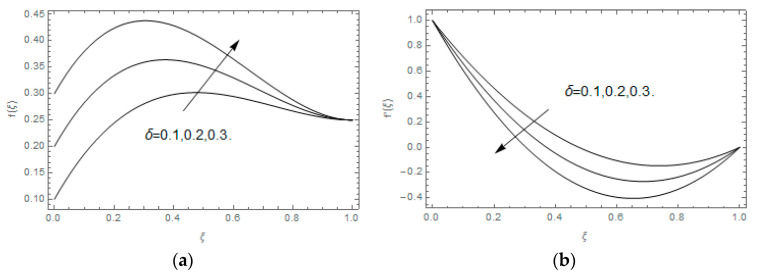
(**a**,**b**): Impression of δ on f(ξ) and $f^{\prime }\left(\xi \right)$.

**Figure 8:**
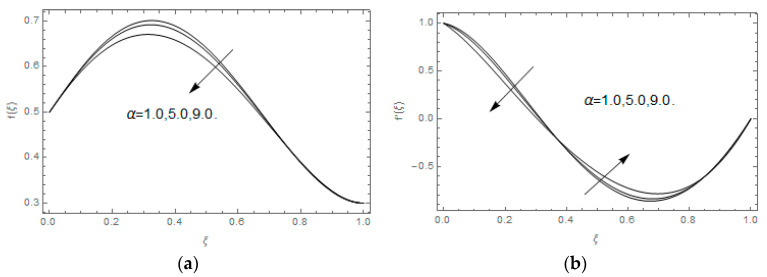
(**a**,**b**): Impression of α on f(ξ) and $f^{\prime }\left(\xi \right)$.

**Figure 9:**
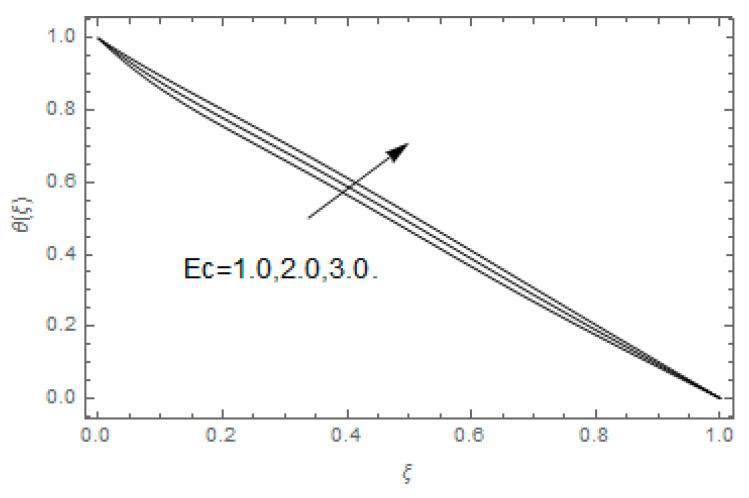
Impression of Ec over θ(ξ).

**Figure 10:**
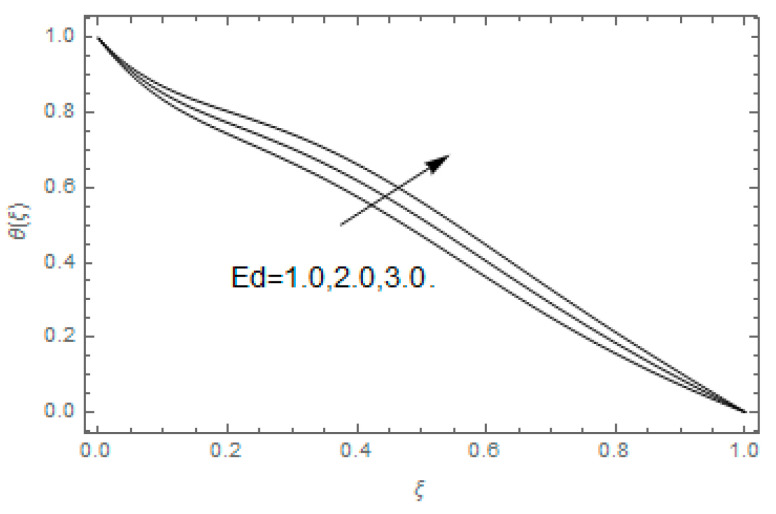
Impression of Ed over θ(ξ).

**Figure 11:**
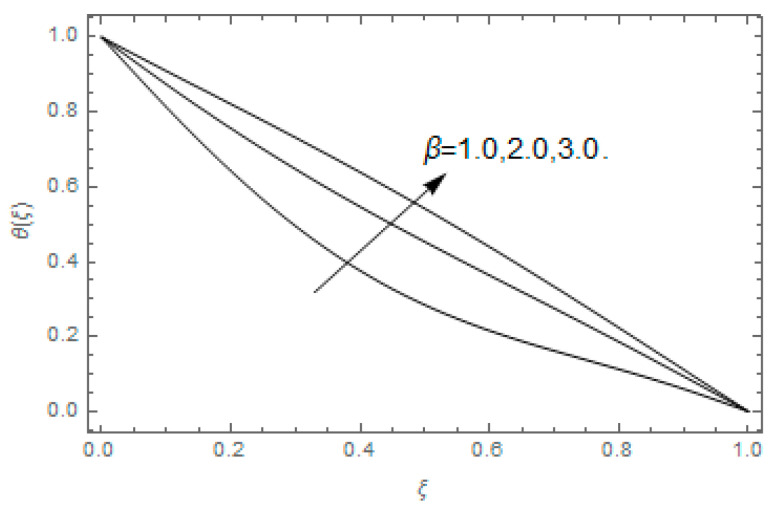
Impression of β over θ(ξ).

**Figure 12:**
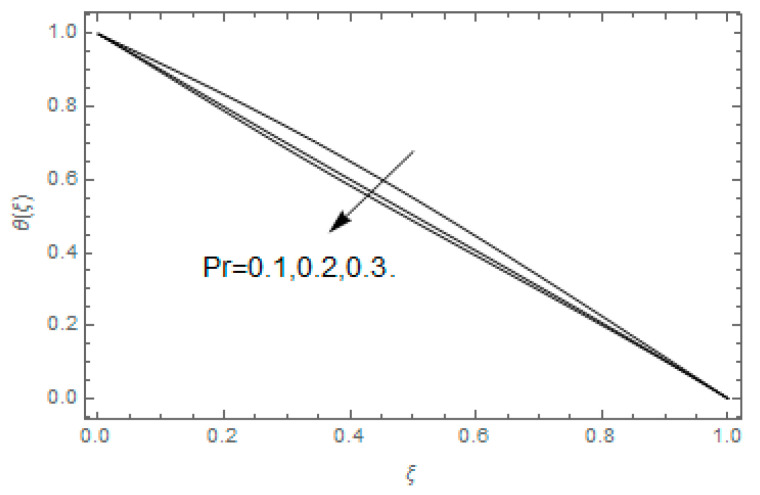
Impact of Pr over θ(ξ).

**Figure 13:**
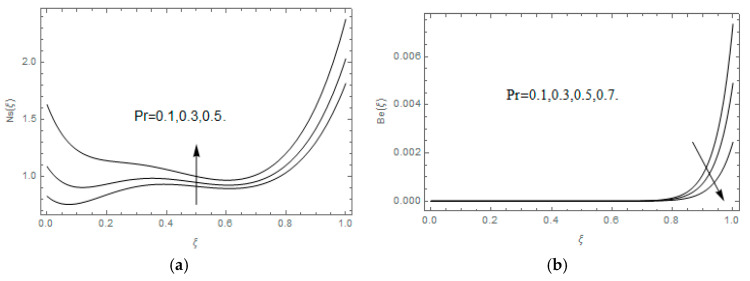
(**a**,**b**): Impression Pr on Ns(ξ) and Be(ξ).

**Figure 14:**
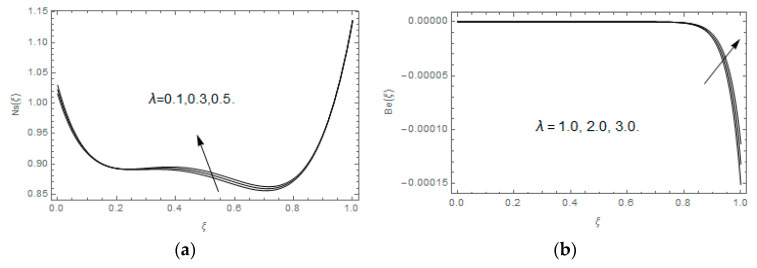
(**a**,**b**): Impression of λ on Ns(ξ) and Be(ξ).

**Figure 15:**
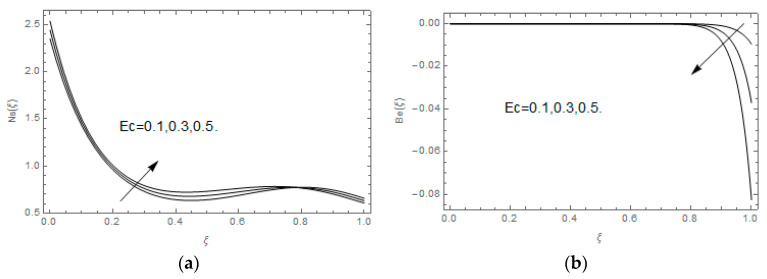
(**a**,**b**): Impression of Ec on Ns(ξ) and Be(ξ).

**Figure 16:**
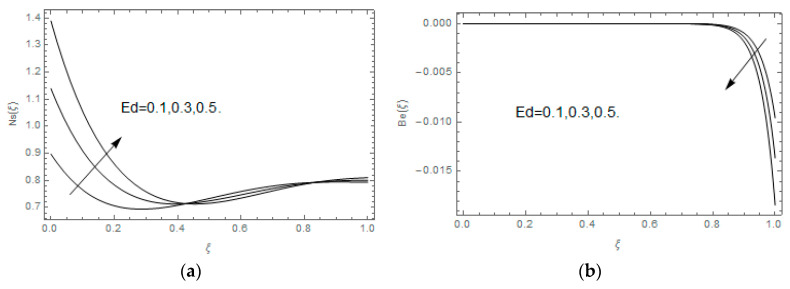
(**a**,**b**): Impression of Ed on Ns(ξ) and Be(ξ).

The impact of Casson parameter $\beta_{o}$ is depicted in Figure 3a,b. The growing Casson parameter declines the velocity profiles $\left(f^{\prime }\left(\xi \right),f\left(\xi \right)\right)$ of the nanofluid flow. Actually, the upsurge in Casson parameter $\beta_{o}$ escalates the plastics dynamic viscosity of the nanofluid, which creates resistance during the flow of nanofluid and decline in the velocity profile occurs. The same impact can be seen in Figure 3b at 0.0≤ξ<0.45. In this figure, the velocity profile increases at 0.45<ξ≤1.0 because of the extending of the lower plate. The impact of porosity parameter λ is depicted in Figure 4a,b. Physically, the porous media acts on the boundary layer flow, which produces the opposition to the fluid’s flow and thus fluid’s velocity declines. This impact is shown in Figure 4a. Similarly, the porosity parameter increases the velocity profile at 0.0≤ξ≤0.40 while it decreases at 0.4≤ξ≤1.0 as shown in Figure 4b. This impact is because of the extending of the lower plate. The impact of squeezing parameter β is portrayed in Figure 5a,b. The escalation in β upsurges the velocity profiles. Physically, the higher values of β move the upper surface descending and additional pressure utilized over fluid’s particles. Therefore, the velocity profiles increased. Figure 6a,b displays the result of M on velocity profiles. Actually, the Lorentz force declares that the induced M resists the fluid motion on the liquid boundary, which as a result, diminishes the velocity of the liquid. The impression of δ on velocity profiles is illustrated in Figure 7a,b. The higher values of δ upturns the velocity along x-direction while the greater values of δ reduce the velocity in y-direction. The impression of rotation α is depicted in Figure 8a,b. Clearly, from Figure 8a, the increase in α reduces the velocity profile in x-direction. From Figure 8b, the velocity profile reduces in y-direction at 0.0≤ξ<0.40 while it increases at 0.4≤ξ≤1.0. The impression of Ec on θ(ξ) is offered in Figure 9. The increasing Ec upsurges the fluid flow temperature. Actually, the Eckert number produces viscous resistance due to the occurrence of dissipation term, which increases the nanofluid thermal conductivity to upsurge the temperature field. A similar impact can be seen in Figure 10. The influence of squeezing β on θ(ξ) is depicted in Figure 11. Unmistakably, we saw that the temperature increases rapidly with the enlargement in β. Bigger β implies that the upper plate moves in a descending fashion in consequence with the fluid interatomic collision increments because of the little space accessible concerning the plates to the nanofluid particles. At the point when the impacts among the atoms of the nanofluid improve, the nanofluid temperature upsurges. Figure 12 exhibits the impression of Pr on θ(ξ). Here, we analyzed that temperature field diminishes by means of escalation in Pr. Bigger estimations of Pr connects to lesser heat conductivity, hence the temperature profile is diminished. Figure 13a illustrates the impression of Pr on Ns(ξ). The value of Pr escalates the entropy generation. The magnetic field boosts the form of the plate surface to its maximum values in the area of the plate. The opposite impact of Prandtl number Pr on Be(ξ) is demonstrated in Figure 1. Figure 14a,b denotes the effect of porosity parameter λ over Ns(ξ) and Be(ξ). Both the entropy and Bejan number boost with the escalation in porous media. It is perceived that the entropy upsurges more speedily compared to Bejan number. Figure 15a,b demonstrates the impact of Eckert number Ec over Ns(ξ) and Be(ξ). The escalation in Eckert number upsurges the entropy generation Ns(ξ) while it diminishes the Be(ξ). Similar impacts of Ed on Ns(ξ) and Be(ξ) are observed in Figure 16a,b.

The impressions of Casson parameter $\beta_{o}$, magnetic field M, rotation parameter α, squeezing parameter β, suction parameter δ, porosity parameter λ, Prandtl number Pr, and local Eckert numbers {Ec, Ed} over skin friction $C_{fx}$ and Nusselt number $Nu_{x}$ are displayed in Table 1 and Table 2, respectively. The escalating approximations of squeezing and rotation parameters reduced the $C_{fx}$ while the rise in Casson, porosity, and magnetic field parameters augmented the $C_{fx}$. The rising approximations of squeezing parameter showed dual behavior in $Nu_{x}$. The escalating approximations of Prandtl number, Eckert numbers, and magnetic field parameter declined the $Nu_{x}$.

## 5. Comparison of HAM with Numerical Result

We solve the system the model Equations (9)–(11) with boundary conditions (12) by ND Solve in Mathematica 10 package. Comparison between analytical and numerical techniques is shown in Table 3 and Table 4. An excellent agreement is fund.

## 6. Conclusions

The squeezing Casson nanofluid flow with viscous dissipation and entropy generation between two parallel stretching plates has been presented in this paper. The stretching plates are considered porous. The impact of magnetic field is also reflected in the proposed model. The impacts of embedded parameters are shown through figures and tables. 

The significant facts of the presented model are as follows:

- The Casson parameter, porosity parameter, magnetic field parameter, and rotation parameter reduced the velocity profile f(ξ) while the squeezing and suction parameters increased the velocity profile f(ξ).
- The Casson parameter, porosity parameter, magnetic field parameter, and rotation parameter showed dual behavior in the velocity profile $f^{\prime }\left(\xi \right)$.
- The squeezing parameter \ increased the velocity profile $f^{\prime }\left(\xi \right)$ while the suction parameter reduced the velocity profile $f^{\prime }\left(\xi \right)$.
- The Eckert numbers and squeezing parameter increased the temperature profile θ(ξ) while the Prandtl number increased the temperature profile θ(ξ).
- The Prandtl number, porosity parameter, and Eckert numbers increased the Entropy generation rate Ns(ξ).
- The porosity parameter increased the Bejan number Be(ξ) while the Eckert and Prandtl numbers increased the Bejan number.

## Figures and Tables

**Table 1:** Impacts of $\beta ,\;\beta_{o},\;\alpha$, and M on $C_{fx}$.

β	$\beta_{o}$	λ	α	M	$C_{fx}$
1.2	0.1	0.1	0.1	0.2	0.955529
1.3					0.344419
1.4					−0.247015
1.2	0.2				0.984942
	0.3				1.009420
	0.4				1.029430
1.2	0.1	0.2			0.956860
		0.4			0.959551
		0.5			0.962230
1.2	0.1	0.1	0.2		0.955522
			0.3		0.955510
			0.4		0.955493
1.2	0.1	0.1	0.1	0.3	0.956887
				0.4	0.958211
				0.5	0.959551

**Table 2:** The impacts of β, Pr, Ec, Ed, and M on $Nu_{x}$.

β	Pr	Ec	Ed	M	$Nu_{x}$
1.2	0.1	0.5	0.6	0.2	0.323922
1.3					0.534419
1.4					0.169332
1.2	0.2				0.256999
	0.3				0.190056
	0.4				0.123293
1.2	0.1				0.056644
		0.6			0.200577
		0.7			0.077723
		0.8			0.046114
1.2	0.1	0.5	0.7		0.313305
			0.8		0.302689
			0.9		0.292072
1.2	0.1	0.5	0.6	0.3	0.292126
				0.4	0.200330
				0.5	0.128534

**Table 3:** Comparison of HAM and numerical results for velocity profile.

ξ	HAM	Numerical	Difference
0.0	0.1	$-6.9368\times 10^{-9}$	1
0.2	0.269142	0.170298	0.098844
0.4	0.383833	0.288010	0.095823
0.6	0.454293	0.362311	0.091982
0.8	0.490022	0.401375	0.088647
1.0	0.5	0.412773	0.087227

**Table 4:** Comparison of HAM and numerical results for temperature profile.

ξ	HAM	Numerical	Difference
0.0	1	1	0.0
0.2	0.799983	0.800563	$5.8\times 10^{-\;4}$
0.4	0.599511	0.599659	$1.48\times 10^{-4}$
0.6	0.399278	0.398728	$5.5\times 10^{-4}$
0.8	0.199484	0.198671	$8.13\times 10^{-4}$
1.0	$1.1102\times 10^{-16}$	$1.5240\times 10^{-9}$	$1.52\times 10^{-9}$

## Nominiclature

Symboles	Description
u,v,w	Velocity Components
x,y,z	Coordinate Axes
P	Pressure
$B_{o}$	Magnetic Field
s	Time Parameter
t	Time
$C_{p}$	Specific Heat Capacity
a	Stretching Parameter
h	Gap between walls/ Plates
T	Temperature
M	Magnetic Parameter
$q_{w}$	Heat Flux
$U_{w}$	Stretching velocity
$\Omega_{o}$	Angular Velocity
$V_{o}$	Suction Velocity
Greek Latters	
α	Rotation Parameter
β	Squeezing Parameter
$\beta_{o}$	Casson Fluid Parameter
δ	Suction Parameter
σ	Electrical Conductivity
τ	Shear Stress
λ	Porosity Parameter
μ	Dynamic viscosity
ν	Kinematic viscosity
ρ	Density
θ	Temperature Profile
Φ	Nanoparticles fraction by Volume
$\kappa^{*}$	Thermal Conductivity
Dimensionless Numbers	
Pr	Prandtl Number
Be	Bejan Number
Ec,Ed	Eckert Numbers
$Nu_{x}$	Nusselt number
Rex	Reynolds Number
$N_{i},i=1,2,3,4,5$	Ratios of Physical Quantities
Subscripts	
nf	Nanofluid
f	Base Fluid
s	Solid Nanoparticles
w	At lower wall/ Plate
h	At Upper wall/ Plate
